# The Germ Theory of Dental Caries

**Published:** 1888-10

**Authors:** George S. Allan

**Affiliations:** New York City


### Independent Practitioner.

Vol. IX. October, 1888. No. 10.

Note.—No paper published or to be published in another journal will be accepted for this department. All papers must be in the hands of the Editor before the first day of the month preceding that in which they are expected to appear. Extra copies will be furnished to each contributor of an accepted original article, and reprints, in pamphlet form, may be had at the cost of the paper, press-work and binding, if ordered when the manuscript is forwarded. The Editor and Publishers are not responsible for the opinions expressed by contributors. The journal is issued promptly, on the first day of each month.

### Original Communications.

### THE GERM THEORY OF DENTAL CARIES.

BY GEO. S. ALLAN, NEW YORK CITY.

Read before the Connecticut Valley and Massachusetts Societies at their Union Meeting, held in Boston, July 10th, 1888.

Dental art has existed for many a long day, but dental science is of far more modern origin. The profession welcomes the latter, and rejoices in its growth and development; for it marks a new era and a higher standing for all that pertains to our specialty. Science is most exacting in its demands and methods of research, and calls into its service the most highly trained minds; and so everything it touches or is interested in is elevated and enriched.

Few professions or callings have advanced more rapidly in the last decade than dentistry; and this is mostly due to the good work science has done for it.

The subject I have to present to you this evening belongs purely to the domain of dental science, and is the latest and most valuable contribution to our literature. Dealing as it does with first causes, it commands our attention, and attracts by the promise it makes of new and more efficient methods of practice.

The etiology of dental caries has, from the beginning, attracted far more than an average amount of attention and thought.

Few results, however, of any value, up to a late date, have rewarded the efforts of those who have attempted the solution of the problem. Theories and beliefs were plenty, but, because of their slight foundation, they commanded little respect. Cleanliness and the filling of cavities already formed with some indestructible material, were known to be valuable means in arresting decay; but why, or wherefore, could not be stated with any certainty. It was strongly suspected that an acid or acids in some way gained access to the oral cavity, and was the active agent or agents in destroying the teeth; but how it got there, or what conditions of health or disease promoted its formation, could only be guessed at, and by many these difficulties were considered so great as to lead them to reject the idea entirely. "Why," they said, "lay the decay of teeth to an acid, when you can neither account for its presence in the mouth, or for its peculiar mode of action in forming well-marked cavities in the teeth instead of, as would naturally be suspected, uniformly acting on the surface of the teeth." Hence the vital and the chemico-vital theory, and a host of others, each having its advocates and followers in proportion as it seemed plausible, or accounted for the conditions presented. No one theory had any great following, for the simple reason that none was free from serious objections. There were too many broken links in the chain of evidence connecting cause and effect. The mixed chemico-vital theory was probably the most strongly entrenched of any; still there were many blots on its character, and few spoke of it with more than doubtful respect. To Dr. W. D. Miller, of Germany, an American I am proud to say, belongs the honor of having solved the problem, and of giving us not only a theory of dental caries, but of demonstrating his theory by so thorough, painstaking and careful a series of experiments as to leave no doubt in the minds of fair men that it is not only the true theory, but the only one resting on a scientific basis. Great credit, therefore, belongs to Dr. Miller for his valuable work. He has placed the profession under many obligations—obligations which I firmly believe they will be only too glad to repay in honor and fame. I do not appear before you to-night in defense of the theory, or because it is not well-grounded, and needing support or vindication; but because it is comparatively new and may not be familiar to all; and, further, because, through the kindness of Dr. Miller, I have had more than the average opportunity of studying it. My hope and desire is to so present it that you may take hold of it in its simplicity and completeness, and see how fully all doubts and objections have been met, and how carefully and thoroughly Dr. Miller has finished the self-imposed task.

In commencing, I hope you will pardon me for giving a short, general description of bacteria; what they are, how they grow and do their work. I will go into detail only sufficient to make my picture fairly complete, and enable those who may be rusty in their reading to follow the chain of evidence and reasoning that led Dr. Miller to his conclusions. In other words, I will try to anticipate such questions as would naturally suggest themselves to the minds of those not well posted if I were showing them the actual specimens under the microscope. A method of instruction which I am most sorry I cannot carry out at the present time, for it would be by far the most satisfactory to pursue. Fortunately, the salient facts in regard to the theory are not many, and are easily grasped; and with the help of the lantern and photographs that I have had prepared, and which are the best substitute, and a fairly good one, for the microscope and slides, I hope to attain a moderate success.

Let me premise that I have nothing specially original to offer you. My observations upon the subject have been mainly based upon some thirty odd preparations kindly sent me by Dr. Miller, and such general work with the microscope on the fluids of the mouth, and the contents of the cavities found in carious teeth, as would naturally follow in seeking such information. It has been a study beset with many difficulties, but compassing many pleasures and no little satisfaction.

He who takes up the study of bacteria enters a veritable wonderland, and the deeper into it he dips, the greater will be his surprise, amazement and delight. He will be amazed to learn how important a role they play in life, and that his very continuance in life depends upon their presence in, or absence from, his system; and he will be delighted with the new world of knowledge presented to him. A new science has been created, that of Mycology, which to-day is exciting more general interest, and engaging more thought and study than most of the older sciences. A new literature of surprising extent and variety is being created, and so eager and numerous are those who occupy their time with it, and so steady is the stream of their discoveries, that we seem to be but on the threshold of what we are to know in the near future.

What then are bacteria? What forms and shapes do they present? How do they grow, increase and multiply? First, then: Bacteria are the smallest of known organisms, and, though near the border line separating animal and vegetable life, are now placed among the plants. For a time their true classification was in doubt; but as the study of their life processes proceeded, it was seen that most of their analogies were with the vegetables. To this view almost all the authorities of note agree. De Barry, vide Lectures on Bacteria, pp. 37: "We can only say, therefore, that the bacteria, together with the rest of the schysophytes, are a group of simple plants of a low order." Bacteria, in brief, are unicellular plants; plants composed of one cell, and that of the simplest character and most minute size. They are formed, mainly, if not entirely, of protoplasm; a cell envelope or wall of more or less consistency is commonly present, but cannot be said to be a universal characteristic. So far as known they are structureless; that is to say that, owing to their extreme minuteness, even by the use of the very highest powers we have failed to differentiate the elements of which they are composed. In size, bacteria differ among themselves greatly; but they are small compared with other vegetable cells. Expressed in fractions of an English inch, they vary from $\frac{1}{100000}$ or less, for many are too small to be measured, to $\frac{1}{125}$ of an inch. In shape and form they vary somewhat, but not as much as might be expected; the simple spherule, or dot you may say, being the most common, and the same elongated in one direction so as to form a rod, coming next. The first are commonly called cocci, the second bacilli, and the same twisted on themselves spirilli. Little, however, can be made of individual shapes, and they cannot, as a rule, in that way be separated into species. The highest powers of the microscope fail to accomplish this. That there are numerous species, and genera even, does not admit of question; but a study of their life history, and their physiological, as well as their morphological, characteristics is necessary to determine them. Bacteria that cannot be distinguished one from the other, in the isolated state, present in cultures striking differences, not only in their groupings, but in the changes they effect in the media or substratum they grow upon.

In the manner of development and propagation, bacteria have many characteristics in common with the higher forms of plant life. The individual cell first elongates in one direction until it is nearly or quite double that of the original cell. Then, after a short period of rest, a slight constriction may be observed in the centre, not of the cell as a whole, but of its contents. This continues until the cell is divided. Sometimes these cells thus formed fall apart, and again they remain united; but in either case they have nothing in common. This process goes on continually. A cell that does not grow soon loses its vitality, and the presumption is that the great mass of bacteria perish in this way. The formation of spores, the second method provided for the continuation of the species, generally commences when the process of multiplication by division ceases. The contents of the cell, instead of dividing, contracts on itself, forming a so-called spore, which afterwards, by the breaking up of the cell wall, is liberated. Commonly but one spore or seed is formed, but at times from four to six may be developed. These spores thus formed present some striking characteristics, the most noticeable of which consists in their pronounced vitality. They resist greater extremes of temperature, stronger acids and alkalies than the parent cells, and in every way are more difficult to destroy.

One of the most interesting features of bacterial life is found in their waste products, and the influences they exert on the media in which they grow. It is not common to think of waste products in connection with plant life; but they are no more a feature of animal life than they are of plant life. The fæces, the urine, the carbon-dioxide from the lungs are the effete matters of animal life, and have their analogies in plant life. In proportion to their size, animals absorb a larger proportion of food products than vegetables, and cast off more, and in a more visible form. But here the difference ceases.

Plants store up large amounts of waste products in their tissues, notably in the woody fibres, the outer layers of the bark, and in the leaves that drop off in the fall, etc. Starch is stored up in the potato. Then again the alkaloids, such as quinine, strychnine, and the gummy exudations, and resins are also waste products. The roots of plants throw off much waste material, but these are mostly absorbed by the soil in which they grow.

One classification made in bacteria relates solely to their habitat, whether it is in a living or a dead organism—the former taking the name of parasites, the latter saprophytes. Pathologists have their hands full in dealing with the former; our business is with the saprophytes only. To this class belong the fermentative, the putrefactive, and the pigment forms and varieties. Zymogenic or ferment-producing bacteria flourish in non-nitrogenized media. The putrefactive bacteria acts on proteids and nitrogenized material generally, and in many ways their action is more complex than is that of the ferments, though analogous to it. The compounds resulting from their growth are oftentimes most offensive and even dangerous to life when taken into the system. The so-called ptomaines belong to this group, and are active poisons, and that in proportion to the dose. They are septic poisons, not septic infections. Whether they belong to the waste products of bacterial life, or are formed by the action of some waste products on the organic menstruum in which they are produced, cannot be stated with certainty.

A most interesting point connected with bacterial life is the conditions which favor their existence, as well as those that antagonise it. But little is known concerning them. The field of investigation here is large and promises most valuable results; for it is in this direction that we must look for means to prevent their ravages. It is easy enough in the laboratory to try the effects of various degrees of temperature, acids, alkalies, etc.; but these experiments tell but little that is useful when applied to the human system, for there they do not repeat themselves. There are other agencies far more subtle in their mode of actions that have not been discovered, that are far more potent in power and mode of action. They are the ones that are constantly promoting and retarding bacterial life. Fortunately, the science being comparatively new, and the number of workers many, it is reasonable to suppose that the near future will bring a solution of the vexed question. It will be a grand gain for suffering humanity when it is reached.

We dentists are more especially interested in the physiological aspect of the question ; and, although we unquestionably know the prime cause of dental caries, we are as yet groping in the dark for means of prevention. Those that we have are crude and cumbersome and do not always hit the mark.

The study of bacteria requires special apparatus to meet definite wants and the liabilities for error are many. There is, probably, no branch of study which calls for greater care, patience and persevering effort, and no one who takes it up need expect good results who has not all these requisites at his command.

One of the first and greatest difficulties which met the early workers in this field was in obtaining "pure cultures;" that is, to say, a growth of one species only, and to maintain them through successive generations, so that the life history of each individual, and all related to it, could be determined. In this way only can results of value and precision be obtained. At first sight it would seem to be an impossible task to pick out individual bacteria and plant them in a medium free from all other bacteria, or the germs of same, and maintain these conditions for any length of time. Many and various were the plans proposed and tried before the desired end was attained. Prof. Koch, of Berlin, however, finally opened the door and in a way most simple and easy. He so prepared gelatine that would liquefy at about a temperature of 30° cent. Into this he placed other food materials suitable for the growth of bacteria, and the whole was then sterilized by heat. The gelatine thus prepared was spread in thin layers on glass slips, and the germs, or material suspected to contain germs, was scattered over it. Afterwards it was allowed to consolidate by lowering the temperature; generally, the bacterial material was scattered over the gelatine by means of a needle point which was first dipped into the material to be examined, and then drawn across and through the gelatin. In this way, it can be easily seen that some germs would be isolated and held in the stiffened mass where they would grow and develop. Afterwards the separate colonies could be transferred to other vessels and more complete study be made. So much for bacteria in general. Now for our special application.

Koch says, that to determine whether any particular micro-organism is the prime cause of any special disease: 1st. "The micro-organism must be found in the blood, lymph or diseased tissues of man or animal suffering from or dead of the disease." 2d. "The micro-organism thus obtained must be isolated and cultivated in suitable media, that is, outside the animal body. These pure cultivations must be carried on through successive generations of the organism." 3d. "A pure cultivation thus obtained must, when introduced into the body of the healthy animal, produce the disease in question." 4th and lastly. "In the inoculated animal the same micro-organism must again be found." Koch, in laying down these laws, had in mind parasitic bacteria only—those that live upon or in the living body and produce certain well known and marked diseases, such as typhoid or scarlet fever, etc. But I quote and will employ them, though the bacteria of dental caries are saprophytes and live on dead organized matter only. I do this for the reason that Dr. Miller asks no favors in demanding your acceptance of his theory. He makes it conform to the same rigid, unyielding laws. Before Dr. Miller commenced his work it was well known that the tubules of carious teeth were much enlarged, broken down, and filled with micro-organisms; but it was not known whether these fungi were the cause or a sequence of the disease. Leber and Rottenstein had long before published the result of their researches, proving the existence of such organisms in carious teeth, and awarding to them a prominent part in the work of tooth destruction. But they surmised far more than they demonstrated, and stopped short, even in their guess work, of the full meaning of the fungi they had found. It was also well known that the oval cavity was a garden rich and rare in the numbers and variety of bacteria growing and flourishing in every part of it, especially in and around the teeth; and, furthermore, that fermentations were continually going on within its wall, and that these fermentations were accompanied, more or less constantly, by the formation of acids.

Just here Dr. Miller starts in with his work. It would neither be possible or advisable in a paper of this character to attempt to follow all of Dr. Miller's experiments and explanations; only enough will be mentioned to give you the groundwork of his theory. Any one who follows his line of argument, and the evidence, as given in detail in his papers published in the INDEPENDENT PRACTITIONER, Vol. V, Nos. 2, 3, 5, 6, and 7, will see that so far as great care and thorough preparation are concerned, nothing was left undone that could be done to avoid errors or misstatements of facts or conclusions; no one was better aware of the almost endless number of agents which combine to vitiate such experiments and the exceeding great care which was necessary in excluding and eliminating all irrelevant factors. As just stated, it was known that fermentation of various kinds and degrees were constantly going on in the mouth; but the nature of the ferment, whether it was an organized or an unorganized one, or both combined, and their relative values had yet to be settled. The initial experiments of Dr. Miller were instituted to determine these points. Ptyalin, an unorganized ferment, is, of course, constantly present in the fluids of the mouth, and its ferment powers were well known, but Ptyalin is destroyed by a heat of 67° cent., while bacteria are but little affected by the same temperature. On the other hand, a half per cent. solution of carbolic acid or a strong solution of ether rendered bacterial life inert. A short series of experiments, based on these two facts, soon proved that the acid forming ferment in the mouth was one capable of reproducing itself, and was, therefore, an organized ferment. The fact that fungi were present in carious tooth substance, as before stated, was already known. The microscope had revealed their presence to many observers; but that it was an acid producing fungi was not known. In these experiments, material was taken not only from the oral fluids, but from the deeper portions of carious teeth, with corresponding results. The same fungus with like reactions was present in both cases. Again, certain fungi not only do not require free access to the oxygen of the air for their life powers, but thrive better when removed from it, and a classification into a ærobic and anærobic bacteria has been based on this fact.

A second series of experiments proved that the fungi under investigation belongs to the anærobic division, and thrived when removed from the air, a point of the greatest interest, showing as it did, that deep down in the bottom of the tooth, away from the air, this fungus could grow, develop and perfect its destructive work. These two series of experiments throw much light on the etiology of the decay of teeth, and account, as nothing else has accounted, for the peculiar manner in which teeth are destroyed. Whenever, either between the teeth, in sulci or pockets, through faulty development a lodgement can be had for food products, a miniature acid manufactory is set up. The acid freed in this manner decomposes at once that portion of the tooth with which it comes in contact, uniting with its lime-salts and forming new combinations, and as decalcification advances, bacteria follow after, throwing out in their growth new waste products in the form of lactic acid. Chemists tell us that nascent acids, those just formed, are most powerful in their actions in seeking new affinities; and here the acid is continually being eliminated, and in its fresh condition brought in contact with the lime-salts of the teeth. The sugar which is ever present in the mouth, in some form or other, is the natural ferment food on which these bacteria thrive, and if by any chance it is not present, the ptyaline of the saliva soon produces it by its action on the starchy elements of our daily food. These two series of experiments would seem to establish the germ theory on a strong basis. We can safely say they make the truth of the theory most probable and plausible; but others follow which are even more convincing and practically close the argument. They are in keeping with the first of the laws laid down by Koch, in considering pathogenic bacteria, exceeding, however, its requirements. The third in the series of experiments was (*a*) to procure a pure culture of the bacteria found in dental caries. This was obtained in the manner briefly described in a previous page of this paper. The mother material used being the deepest portion of the decayed tooth carefully removed from its bed with a sterilized knife point. (*b*) A little of this pure culture thus obtained was placed in two tubes, the one containing a fermentable mixture, beef extract, to which two per cent. of cane sugar had been added, and the other a non-fermentable fluid, beef extract without sugar. Both tubes and their contents having been previously completely sterilized. Into each tube was placed small pieces of a freshly drawn, healthy bicuspid tooth, also completely sterilized. The solution in the first tube became acid in a few hours; that in the second, not being fermentable, remained neutral. The sections of sound tooth in the first tube soon softened and at the end of a week were pliable and could be bent like pieces of soft cartilage. At the end of the second week all but the thicker sections were completely decalsified, and could be easily cut with a knife. Thin sections of these softened pieces of teeth were then made by means of a freezing microtome. The sections were then stained and mounted in the usual manner, and submitted to microscopic examination. All the well-known signs of dental caries were found to be present; namely, the distended tubules, broken down and distorted by the micro-organisms. Artificially produced caries thus became an accomplished fact. If you ask what became of the pieces of dentine placed in the non-fermentable mixture, the answer is, they suffer no change. No acid being formed, their integrity was not affected. Bear in mind, please, that in the first tube, the one in which the sound pieces of tooth were placed, and afterwards found to be destroyed or in process of destruction, depending on the length of time they were left in the mixture, contained no trace of acid when it was sealed up, and none could gain access to it except such as was formed as a sequence to the fermentation set up by the bacteria. These last experiments would seem to be conclusive to any fair mind, and fully meet the requirements called for by Koch in his last three laws.

A study of the beautifully prepared slides of Dr. Miller are most satisfactory and instructive, and I doubt whether the most expert microscopist could by such an examination alone say whether he was looking at a piece of natural or artificially induced caries, so closely do they simulate each other in all physical characteristics.

Three separate ferments are produced by the fungi of dental caries. The first, an inverting ferment; one capable of changing a non-fermentable sugar, like cane sugar, into a fermentable one, and possibly of forming sugar from starch. The second, an acid producing ferment, the product of which has been proven to be lactic acid. The third, a peptonizing or digestive ferment. All play important parts in the work of tooth destruction. The first prepares the food for the second. The second dissolves out the lime salts of the tooth, and leaves it an easy prey for the third, which, in turn, eats up, dissolves, the animal basis left after the mineral matter has been removed, and reduces the whole to a pulpy or fluid condition.

It would seem unnecessary, before such an audience as this, to take even a moment in pointing out the lession all this teaches. If cleanliness is next to Godliness anywhere, it is in the mouth. Every means should be used to keep it in a state of purity. The use of the tooth-pick, floss-silk and tooth-brush must be constant and thorough. Antiseptics and germicides in some form are also strongly indicated. But just here we find ourselves in a sea of doubt and uncertainty. Germicides and antiseptics we have in plenty, but what ones can be used with safety in the mouth, or which ones possess the most valuable properties remains to be settled. Listerine so far seems to meet the demands most satisfactorily. But it may be set down as a certainty, that no antiseptics will ever make the use of the brush, pick and silk unnecessary. The great want is to find some means to make tooth structure impervious to bacterial invasion, which can only be done by so changing its character that bacteria will not grow in or upon it. So soon as we can, by some application or other methods of treatment, render danger points in teeth such as fissures, sulci and the margins of fillings an unfit soil for their growth, the task will be accomplished.

In office practice bacterial studies, in general, mean a great deal for the safety and comfort of our patients, and the dentist's satisfaction in obtaining good results. In the treatment of dead teeth it insures an amount of success never before thought of. I seldom now use a broach or nerve instrument without first dipping it in a solution of bi-chloride of mercury, of a strength of about one-fifth or one-half of one per cent. In old dead teeth from which the pulps have not been removed, and which heretofore have been so much trouble to treat, by the intelligent use of antiseptics and germicides, treatment becomes easy and comfortable to both patient and operator. The old story we have all listened to so often, "Doctor, that tooth never gave me a particle of trouble till you touched it," ceases now to be a dread.

Knowing whence our trouble comes, and its remedy, we can prevent it. Dr. J. Morgan Howe, of New York, in a most able paper read before the New York State Dental Society at their annual meeting in 1887, pointed out the cause and prevention of this whole class of difficulties. In that paper he suggested the fact that the devitalized pulp was a most favorable basis for bacteria to grow and develope in, and that the long chapter of peridental troubles, that we are so familiar with, had their origin in the entrance of these fungi into the pulp-chamber, and their development in the dead pulp. His remedy consists in making an application of iodoform to the dead pulp, so soon as it is uncovered; and in this way, by making it an unfit soil for the growth of the germs the evil is warded off. I have for sometime shaped my practice in a similar way, using, however, iodol and mercury bi-chloride instead of iodoform. Dr. Howe, I speak from memory, advises leaving the iodoform in the pulp-cavity two or three days, changing daily.

In cleansing teeth, removing tartar, etc., especially in the treatment of pyorhea alveolaris, I dip my instrument constantly in the same bi-chloride mixture. By this means, I am certain to carry it well under the margin of the gums, and into the recesses of the tartar pockets. The results have been most satisfactory.

Every now and then some one, no matter how careful he may be, will expose a pulp. Nowhere do I believe that antiseptic surgery is more valuable and certain in its beneficial effects. In all such cases, I immediately cover the point of exposure with a bit of cotton saturated with mercury bi-chloride solution, or a little powdered iodol. I wish I could tell you which was the better treatment. I am certain that we need fear little trouble from an exposed healthy pulp, if we adopt antiseptic treatment in time.

In all flesh wounds, in the mouth, the same treatment is indicated. No tooth should be extracted, especially in a foul mouth, without following it with an antiseptic application. The same may be said of lancing the gums, opening abscesses, etc.

How far a foul mouth may effect the general health, independent of its action on the teeth, is only commencing to attract attention. Dr. Miller has taken up the study, and already has pointed out many dangers, showing as he does how pathogenic bacteria may in this way gain access to the stomach, lungs, and through abrasions, enter the circulatory system.

In closing, I have a pleasant duty to perform in acknowledging my indebtedness to my good friend, Dr. Andrews. From the first, he has taken the deepest interest in the matter, and voluntarily offered to make such photo-lantern slides as I should need for illustrations. How well he has succeeded, you will soon be able to judge for yourselves; but you cannot know, as I know, the time and patience he has devoted to the task. Had I been aware of what he was undertaking, I never should have allowed him to have undertaken it. When I found out what his self-imposed task meant, I did my best to have some pictures made by other experts, but they all failed. I want also to thank Dr. Sudduth for his assistance and encouragement.

